# Interdisciplinary Management and Provisional Rehabilitation of Severe Anterior Dentoalveolar Trauma Following a Motorcycle Accident in an Adolescent Patient: A Case Report

**DOI:** 10.7759/cureus.112773

**Published:** 2026-07-16

**Authors:** Allan E Sanabria Marcia

**Affiliations:** 1 Dentistry, Excel Dental, Lynn, USA

**Keywords:** adolescent trauma, alveolar ridge preservation, bone grafting, dentoalveolar trauma, emergency dentistry, interdisciplinary care, motorcycle accident, multiple tooth avulsion, temporary fixed bridge, traumatic dental injuries

## Abstract

Traumatic avulsion of multiple anterior teeth is a dental emergency that requires prompt diagnosis, immediate stabilization, and coordinated multidisciplinary management to optimize both immediate and long-term outcomes. This case report describes the comprehensive management of a 15-year-old female patient who presented to a private dental office immediately following a motorcycle accident with complete avulsion of the maxillary anterior teeth (#7, #8, #9, and #10), extensive soft tissue injury, active hemorrhage, and exposed maxillary alveolar bone. Initial treatment focused on emergency assessment, hemorrhage control, clinical and radiographic evaluation to exclude maxillofacial fractures, and screening for signs of associated head injury requiring medical referral. During the emergency procedure, a traumatically exposed impacted maxillary left canine was identified and surgically removed because of the loss of its osseous and soft tissue coverage and its unfavorable prognosis for preservation. Alveolar ridge preservation with particulate bovine bone grafting and primary soft tissue closure was subsequently performed to preserve the remaining hard and soft tissue architecture and facilitate future rehabilitation. Following initial stabilization, the patient was referred to the hospital for a comprehensive medical evaluation, where no additional traumatic injuries were identified. Clinical follow-up demonstrated uneventful healing and progressive soft tissue recovery. Four weeks after the injury, a conservative temporary fixed bridge was fabricated to restore esthetics, function, and patient confidence while preserving adjacent healthy teeth until definitive implant-supported rehabilitation could be performed after completion of craniofacial skeletal growth. This case demonstrates that successful management of severe dentoalveolar trauma extends beyond emergency treatment alone. A systematic, evidence-based clinical approach, interdisciplinary collaboration, preservation of hard and soft tissues, and patient-centered treatment planning were fundamental to achieving favorable functional, esthetic, and long-term outcomes. The clinical principles presented in this report may assist dental practitioners in recognizing, stabilizing, and managing similar traumatic emergencies encountered in everyday clinical practice.

## Introduction

Traumatic dental injuries represent a significant cause of oral morbidity worldwide and are associated with substantial functional, esthetic, and psychosocial consequences, particularly among children and adolescents [1]. Complete avulsion of permanent teeth is one of the most severe dental emergencies because it is frequently accompanied by extensive soft tissue injury, alveolar bone damage, hemorrhage, and significant esthetic impairment, requiring prompt diagnosis and appropriate management to optimize clinical outcomes [2-4].

Comprehensive evaluation of severe dentoalveolar trauma should include clinical and radiographic assessment, hemorrhage control, recognition of associated craniofacial or neurologic injuries requiring medical referral, and timely surgical management when indicated [5-7]. Preservation of the alveolar ridge following traumatic tooth loss is an important component of treatment because maintaining hard and soft tissue architecture facilitates future implant-supported rehabilitation [8-10].

Traumatic injuries may occasionally expose impacted teeth that require individualized surgical decision-making based on the extent of tissue destruction, contamination, and long-term prognosis [11,12]. In adolescent patients, definitive implant placement is generally postponed until completion of craniofacial skeletal growth, making provisional rehabilitation an essential component of treatment planning [13,14]. Because severe anterior tooth loss can also have profound psychological consequences, successful management requires close interdisciplinary collaboration to restore both function and esthetics while supporting the patient's overall well-being [15-17].

This case report describes the interdisciplinary management of a 15-year-old female patient who sustained severe anterior maxillary dentoalveolar trauma following a motorcycle accident, resulting in avulsion of four permanent maxillary incisors and traumatic exposure of an impacted maxillary canine. The educational value of this case lies in the integration of emergency assessment, surgical removal of the traumatically exposed impacted canine, immediate alveolar ridge preservation, timely medical referral, and provisional rehabilitation while considering ongoing craniofacial growth. These clinical decision-making principles may assist clinicians in managing similarly complex traumatic dental injuries [18].

## Case presentation

Patient information

A 15-year-old healthy female (American Society of Anesthesiologists Physical Status Classification I (ASA I)) presented to a private dental office immediately following a motorcycle accident with severe dentoalveolar trauma involving the anterior maxillary region. The patient reported intense pain and active oral bleeding following the accident. Her medical history was noncontributory, and she reported no known allergy to penicillin. Written informed consent for treatment was obtained from the patient's parent or legal guardian before the procedure.

Clinical findings

Clinical examination revealed complete avulsion of the maxillary right lateral incisor (tooth #7), maxillary right central incisor (tooth #8), maxillary left central incisor (tooth #9), and maxillary left lateral incisor (tooth #10), resulting in extensive soft tissue laceration, exposed maxillary alveolar bone, and active hemorrhage (Figure 1).

**Figure 1:**
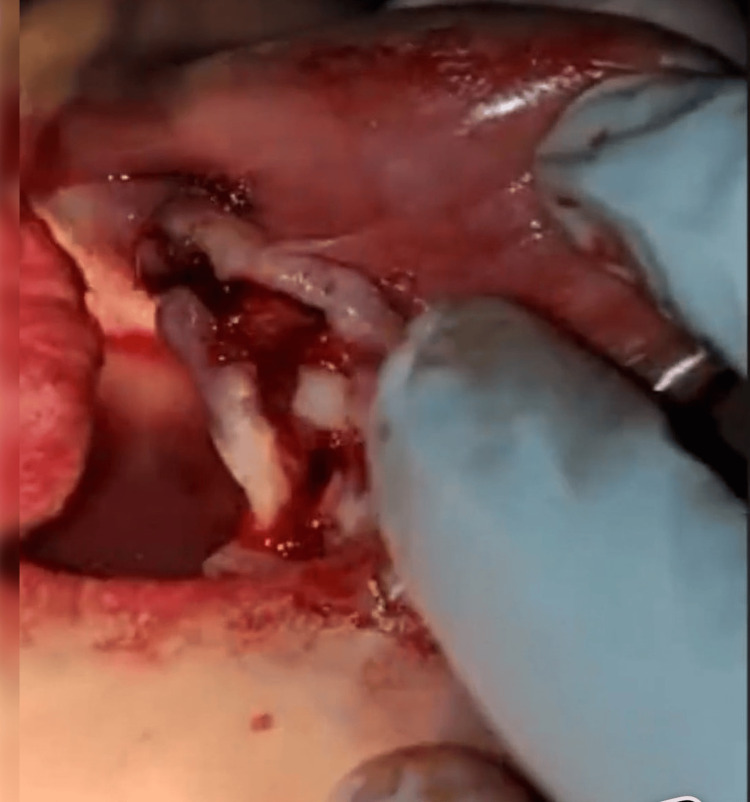
Initial clinical presentation and emergency surgical exploration of the traumatic dentoalveolar injury. Initial intraoral clinical photograph demonstrating complete avulsion of the maxillary anterior teeth, extensive soft tissue laceration, exposed alveolar bone, and active hemorrhage following a motorcycle accident. Surgical exploration confirmed the absence of avulsed teeth within the traumatic wound or alveolar sockets before definitive surgical management.

Initial management focused on emergency stabilization of the patient. The traumatized area was carefully irrigated with sterile saline to remove blood clots, debris, and contaminants, while direct pressure was applied to achieve adequate hemostasis. Because traumatic dental injuries may be associated with head and facial injuries, a rapid medical assessment was performed. The patient was questioned regarding dizziness, nausea, vomiting, loss of consciousness, excessive drowsiness, blurred vision, and other symptoms suggestive of concussion or neurologic injury. She denied experiencing any of these symptoms and reported only severe localized pain in the anterior maxillary region.

Diagnostic assessment

Following the initial clinical assessment and emergency stabilization, a panoramic radiograph was obtained to evaluate the extent of the dentoalveolar injury and exclude associated fractures of the maxilla, mandible, and temporomandibular joints (Figure 2).

**Figure 2:**
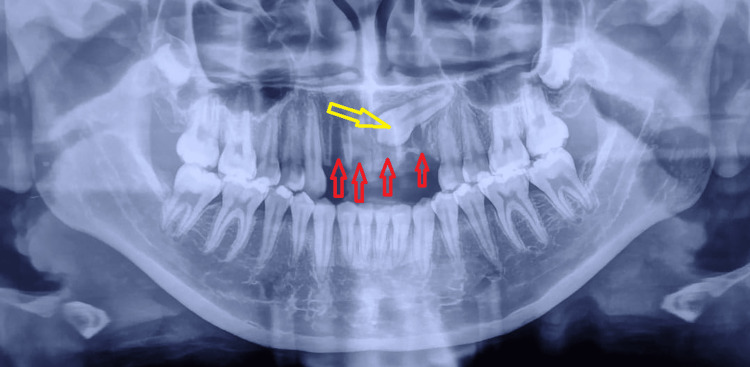
Initial panoramic radiograph obtained immediately after the motorcycle accident. Initial panoramic radiograph obtained immediately after the motorcycle accident. Red arrows indicate avulsion of the maxillary right lateral incisor (tooth #7), maxillary right central incisor (tooth #8), maxillary left central incisor (tooth #9), and maxillary left lateral incisor (tooth #10), with corresponding empty alveolar sockets. The yellow arrow identifies the impacted left maxillary canine. No radiographic evidence of maxillary, mandibular, or condylar fractures was observed.

Radiographic examination confirmed the avulsion of teeth #7, #8, #9, and #10 without evidence of maxillary, mandibular, or condylar fractures. No additional dentoalveolar fractures requiring surgical fixation were identified. Careful clinical and radiographic evaluation also revealed an impacted left maxillary canine that had become traumatically exposed because of the disruption of the overlying alveolar bone and gingival tissues.

Therapeutic intervention

Considering the traumatic exposure of the impacted left maxillary canine, complete loss of its osseous and soft tissue coverage, direct communication with the traumatic wound, and the unfavorable prognosis for predictable preservation, the decision was made to surgically remove the tooth during the emergency procedure (Figure 3).

**Figure 3:**
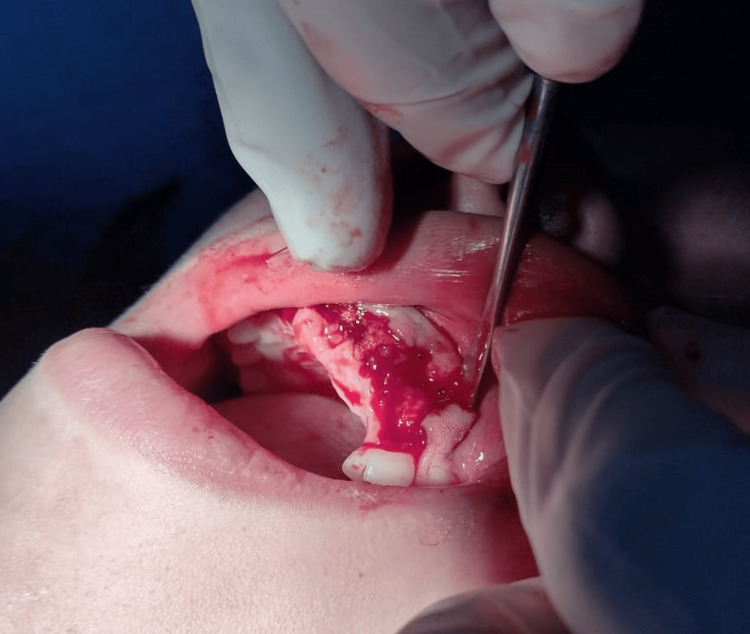
Surgical removal of the traumatically exposed impacted left maxillary canine. Intraoperative clinical photograph demonstrating surgical removal of the traumatically exposed impacted left maxillary canine following severe anterior dentoalveolar trauma. Because of the loss of supporting hard and soft tissues and its unfavorable prognosis, the tooth was extracted as part of the emergency surgical management before alveolar ridge preservation and primary wound closure.

Extraction facilitated thorough debridement of the injured site, reduced the potential risk of infection and delayed wound healing, and enabled adequate alveolar ridge preservation and primary soft tissue closure, thereby optimizing future prosthetic rehabilitation. The extracted impacted left maxillary canine is shown immediately following surgical removal (Figure 4).

**Figure 4:**
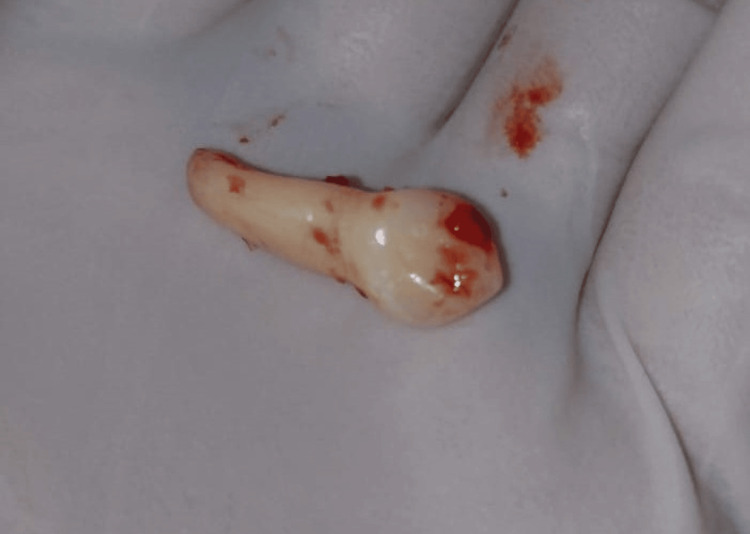
Extracted impacted left maxillary canine following traumatic dentoalveolar injury. The extracted impacted left maxillary canine following severe anterior dentoalveolar trauma. Because of traumatic exposure, loss of supporting hard and soft tissues, and an unfavorable prognosis for preservation, the tooth was surgically removed as part of the emergency management before alveolar ridge preservation and primary wound closure.

Following extraction, particulate deproteinized bovine bone mineral (DBBM) (techBiomat bone, Technology in Biomaterials S.L., Barcelona, Spain) was placed within the traumatic defect and covered with a resorbable collagen membrane to preserve the alveolar ridge and minimize post-traumatic alveolar ridge resorption, thereby maintaining adequate hard tissue volume for future definitive rehabilitation. The traumatized soft tissues were then carefully repositioned and approximated using 3-0 chromic gut sutures to achieve primary wound closure and promote uneventful healing (Figure 5).

**Figure 5:**
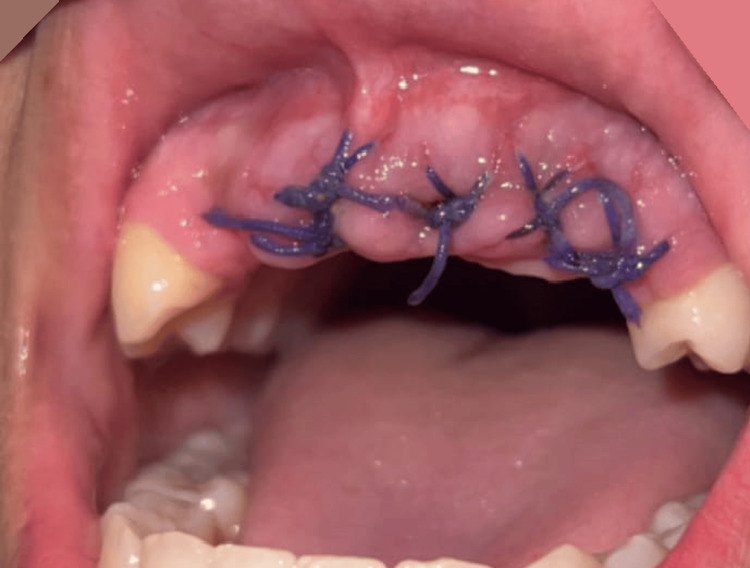
Completion of emergency surgical management with alveolar ridge preservation and primary wound closure. Immediate postoperative intraoral photograph demonstrating completion of the emergency surgical management. The traumatic alveolar defect was grafted with deproteinized bovine bone mineral (DBBM), covered with a resorbable collagen membrane, and closed with 3-0 chromic gut sutures to preserve the alveolar ridge and promote favorable healing for future implant-supported rehabilitation.

The patient was prescribed amoxicillin 500 mg orally three times daily for seven days and ibuprofen 600 mg every eight hours for five days. Although no clinical or radiographic evidence of facial fractures or neurologic impairment was identified during the dental evaluation, the patient was referred to the hospital with the accompanying panoramic radiograph for a comprehensive medical assessment to exclude additional craniofacial or systemic injuries associated with the motorcycle accident.

Follow-up and outcomes

At the one-week postoperative follow-up, the patient reported significant improvement in pain and overall comfort. Clinical examination demonstrated satisfactory soft tissue healing without signs of infection or postoperative complications. Hospital evaluation confirmed the absence of additional traumatic injuries requiring further intervention. During the subsequent two- and four-week postoperative follow-up visits, the resorbable sutures gradually dissolved, postoperative edema resolved, and soft-tissue healing progressed uneventfully (Figure 6).

**Figure 6:**
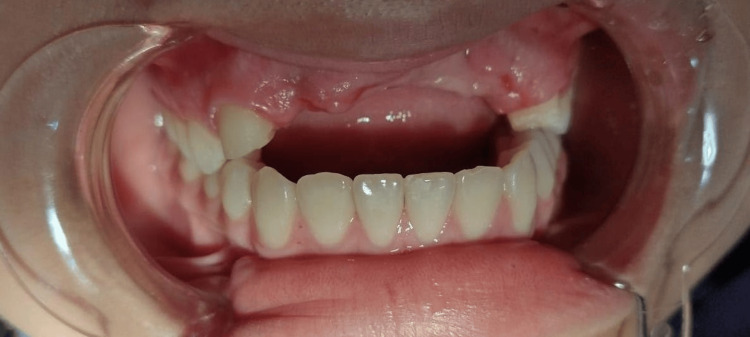
One month postoperative healing and preparation of teeth #6 and #12 for provisional fixed partial denture. Intraoral clinical photograph obtained one month after emergency surgical management demonstrating satisfactory soft tissue healing and preservation of the edentulous maxillary anterior ridge. Following favorable healing, teeth #6 and #12 were prepared as full-coverage abutments for the fabrication of a provisional fixed partial denture.

Four weeks after the initial injury, the surgical site demonstrated excellent soft-tissue healing and satisfactory tissue maturation, creating favorable conditions to begin prosthetic rehabilitation. Because the patient was only 15 years old and craniofacial skeletal growth had not yet been completed, definitive implant placement was postponed in accordance with current recommendations for growing patients. Following confirmation of satisfactory soft tissue healing, prosthetic rehabilitation was initiated. Teeth #6 and #12 were prepared as full coverage abutments for a provisional fixed partial denture. Two weeks later, following laboratory fabrication, the provisional restoration was delivered (Figure 7).

**Figure 7:**
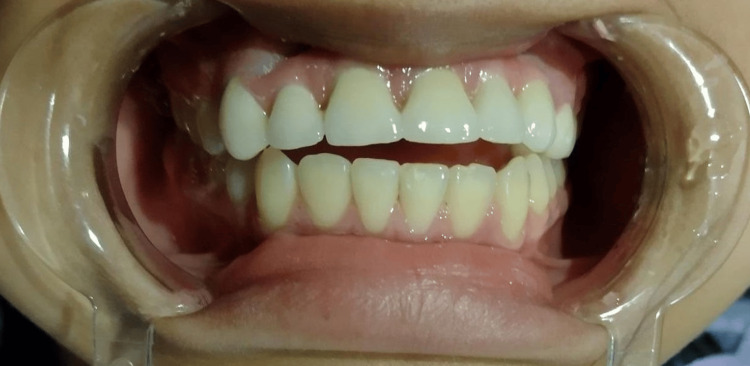
Provisional fixed prosthetic rehabilitation following traumatic dentoalveolar injury. Frontal intraoral photograph demonstrating the provisional fixed partial denture delivered following satisfactory soft tissue healing. The restoration, supported by full coverage abutments on teeth #6 and #12, restored esthetics, phonetics, and masticatory function while maintaining the edentulous space until definitive implant-supported rehabilitation could be performed after completion of craniofacial skeletal growth.

The provisional fixed partial denture restored esthetics, phonetics, and masticatory function, significantly improving the patient's smile, self-confidence, and psychological well-being while maintaining the edentulous space until definitive implant-supported rehabilitation could be performed after completion of skeletal growth (Table 1).

**Table 1:** Timeline

Time	Clinical event and management
Day 0	Patient presented immediately following a motorcycle accident with severe anterior maxillary dentoalveolar trauma, complete avulsion of teeth #7, #8, #9, and #10, extensive soft tissue laceration, exposed alveolar bone, and active hemorrhage.
Day 0	Emergency assessment, hemorrhage control, irrigation of the traumatic wound, and clinical evaluation were performed. Neurologic screening revealed no signs or symptoms of head injury.
Day 0	Panoramic radiograph confirmed avulsion of teeth #7–#10, absence of maxillary, mandibular, or condylar fractures, and identified a traumatically exposed impacted left maxillary canine.
Day 0	Surgical removal of the traumatically exposed impacted left maxillary canine was performed because of its unfavorable prognosis.
Day 0	Alveolar ridge preservation was completed using deproteinized bovine bone mineral (DBBM) and a resorbable collagen membrane, followed by primary wound closure with 3-0 chromic gut sutures.
Day 0	Amoxicillin and ibuprofen were prescribed, and the patient was referred to the hospital for a comprehensive medical evaluation.
1 week	Clinical follow-up demonstrated satisfactory soft tissue healing without signs of infection or complications. Hospital evaluation confirmed the absence of additional traumatic injuries.
2–4 weeks	Progressive uneventful healing, resolution of edema, and gradual resorption of the sutures were observed.
4 weeks	Following satisfactory healing, teeth #6 and #12 were prepared as abutments for a provisional fixed partial denture.
6 weeks	The provisional fixed partial denture was delivered, restoring esthetics, phonetics, and masticatory function while maintaining the edentulous space until definitive implant-supported rehabilitation after completion of craniofacial skeletal growth.

## Discussion

Traumatic avulsion of multiple permanent anterior teeth represents one of the most challenging dental emergencies because successful management requires immediate clinical judgment, coordinated interdisciplinary care, and preservation of future treatment options [1]. In addition to the loss of teeth, these injuries are frequently associated with soft tissue lacerations, alveolar bone defects, contamination of the wound, and significant psychological distress, particularly in adolescent patients [2]. Epidemiological studies have demonstrated that adolescents are among the populations most frequently affected by traumatic dental injuries, particularly following high-energy accidents [3]. The primary objective during the emergency phase is not only to control pain and hemorrhage but also to establish a comprehensive treatment strategy that optimizes both immediate stabilization and long-term functional and esthetic rehabilitation [4].

The initial evaluation of patients with severe dentoalveolar trauma should always extend beyond the oral cavity. Appropriate photographic documentation and radiographic assessment are essential for diagnosis, treatment planning, communication, and long-term follow-up [5]. Screening for neurologic symptoms such as dizziness, nausea, vomiting, altered consciousness, visual disturbances, and excessive drowsiness is an essential component of the emergency assessment [6]. In cases involving severe dentoalveolar trauma, surgical intervention may be necessary to remove nonviable tissues, manage associated alveolar injuries, and establish favorable conditions for future oral rehabilitation [7].

Preservation of the alveolar ridge following traumatic tooth loss is an important component of long-term treatment planning because post-traumatic bone remodeling may compromise future implant placement and esthetic outcomes [8]. Ridge preservation using particulate bone grafting helps maintain alveolar volume, minimizes ridge collapse, and creates favorable conditions for future implant-supported rehabilitation [9]. Nevertheless, ridge preservation cannot completely prevent post-traumatic bone remodeling, particularly in traumatic defects, where the extent of osseous and soft-tissue injury may influence healing and the final ridge dimensions. Comprehensive management of traumatic dental injuries frequently requires coordinated surgical and restorative treatment planning to optimize both functional and esthetic outcomes [10].

In the present case, panoramic imaging confirmed the absence of maxillary, mandibular, and condylar fractures while simultaneously identifying a traumatically exposed impacted maxillary canine. The decision to remove the impacted tooth was based on traumatic exposure, loss of its protective osseous and soft tissue coverage, increased risk of bacterial contamination, and the potential interference with wound healing and future prosthetic rehabilitation [11]. Surgical management of impacted maxillary canines should always be individualized according to the clinical presentation, with treatment decisions based on the condition of the surrounding hard and soft tissues and the long-term prognosis of the tooth [12].

Treatment planning in adolescent patients requires special consideration because implant placement is generally postponed until completion of craniofacial skeletal growth to minimize the risk of infraocclusion and unfavorable esthetic outcomes associated with continued maxillary development [13]. Consequently, appropriate interim restorative options should be selected according to the patient's age, clinical condition, and long-term treatment plan. Although removable partial dentures and adhesive resin-bonded fixed partial dentures are recognized interim treatment options, they were considered less suitable in the present case because of the extensive four-tooth anterior edentulous span and the need to provide predictable esthetics, phonetics, function, and patient comfort following severe trauma. Therefore, a provisional fixed partial denture supported by full coverage abutments on teeth #6 and #12 was selected to provide greater stability during healing while maintaining the edentulous space for future definitive implant-supported rehabilitation after completion of craniofacial skeletal growth [14].

Beyond the surgical and restorative aspects of treatment, severe anterior dental trauma has profound psychosocial consequences. The unexpected loss of multiple anterior teeth during adolescence may significantly affect self-esteem, social interactions, speech, and overall quality of life [15]. Successful management, therefore, requires close interdisciplinary collaboration among dental and medical professionals to coordinate emergency treatment, surgical care, postoperative follow-up, and definitive rehabilitation [16].

Because this patient's injuries resulted from a motorcycle accident, a comprehensive medical evaluation was essential to exclude associated maxillofacial and systemic injuries that frequently accompany high-energy trauma [17]. The favorable healing observed throughout follow-up further emphasizes that a systematic approach integrating emergency assessment, evidence-based surgical management, preservation of hard and soft tissues, conservative interim rehabilitation, and long-term follow-up can produce excellent functional, esthetic, and psychological outcomes in patients with severe traumatic dental injuries [18].

## Conclusions

This case illustrates the importance of a structured and systematic approach to the management of severe dentoalveolar trauma. Although based on a single patient, it highlights important clinical considerations in the emergency assessment, surgical management, and provisional rehabilitation of complex traumatic dental injuries. Maintaining composure during emergency situations, adhering to established trauma principles, prioritizing patient safety, recognizing the need for timely medical referral, preserving hard and soft tissues, and working effectively within an interdisciplinary team are fundamental components of comprehensive trauma care.

The successful management of traumatic dental injuries extends beyond replacing missing teeth. Careful emergency assessment, sound surgical decision making, appropriate radiographic evaluation, alveolar ridge preservation when indicated, conservative provisional rehabilitation, and long-term treatment planning are all important aspects of patient-centered care. In adolescent patients, consideration of ongoing craniofacial skeletal growth remains essential when selecting definitive treatment options.

This case provides practical clinical insights into the management of severe anterior dentoalveolar trauma and may assist clinicians when encountering similar complex presentations. While the treatment approach described should not be generalized beyond the context of this individual patient, the clinical considerations highlighted in this report may contribute to informed clinical decision-making and interdisciplinary management in comparable situations.
